# Erythropoietin (EPO) in acute kidney injury

**DOI:** 10.1186/2110-5820-1-3

**Published:** 2011-03-21

**Authors:** Elizabeth Moore, Rinaldo Bellomo

**Affiliations:** 1 Australian and New Zealand Intensive Care Research Centre, Department of Epidemiology and Preventive Medicine, School of Public Health and Preventive Medicine, Monash University, The Alfred Centre, 99 Commercial Road, Melbourne, VIC 3004, Australia

## Abstract

Erythropoietin (EPO) is a 30.4 kDa glycoprotein produced by the kidney, and is mostly well-known for its physiological function in regulating red blood cell production in the bone marrow. Accumulating evidence, however, suggests that EPO has additional organ protective effects, which may be useful in the prevention or treatment of acute kidney injury. These protective mechanisms are multifactorial in nature and include inhibition of apoptotic cell death, stimulation of cellular regeneration, inhibition of deleterious pathways, and promotion of recovery.

In this article, we review the physiology of EPO, assess previous work that supports the role of EPO as a general tissue protective agent, and explain the mechanisms by which it may achieve this tissue protective effect. We then focus on experimental and clinical data that suggest that EPO has a kidney protective effect.

## Introduction

Erythropoietin (EPO) is a complex molecule, which regulates red blood cell production in the bone marrow. Recombinant human EPO (rHuEPO) is commercially available and is widely used for the treatment of anemia. In recent years, additional nonerythropoietic tissue/organ protective properties of EPO have become apparent, in particular for kidneys. In this article, we consider the evidence supporting EPO as a general tissue protective drug and discuss the potential mechanisms by which it may achieve this general effect. We then focus on the renal protective effects of EPO and the potential mechanisms through which it may confer this specific protection. Finally, we review the experimental studies and clinical trials of EPO in acute kidney injury (AKI)--discuss risks, lessons learned, and the need for further randomized studies in humans before any change in clinical practice is considered.

## The physiological properties of EPO

### The structure of the EPO molecule

EPO is a 30.4 kD glycoprotein and class I cytokine consisting of 165 amino acids [1]. EPO has four acidic oligosaccharide side chains (3 N-linked and 1 O-linked) and contains up to 14 sialic acid residues. Its carbohydrate portion contributes 40% of its molecular weight [1]. The N-linked polysaccharide side chains appear to be important for the biosynthesis and secretion of EPO, enhance its stability in blood, and limit hepatic clearance, thus facilitating the systemic transit of EPO from kidney to bone marrow [2].

The variable nature of the sialic acid content gives rise to EPO isoforms with differences in charge. As the number of sialic acid groups on the carbohydrate portion of EPO increase, so does its serum half-life, whereas receptor-binding capacity decreases [3-7]. Clearance, however, appears to have a stronger influence on *in vivo *activity than receptor-binding affinity.

**Table 1:** Animal studies of EPO in AKI: ischemia-reperfusion models

Year	Type	AKI model/follow-up	EPO dose	Outcome
2009	Dogs	IR: nephrectomy, 2 weeks recovery, then renal artery occlusion, ischemia 1 hr; reperfusion 28 days	EPO 500 U/kg i.v. before ischemia ± 90 min abdominal insufflation	↓microalbuminuria, ↑renal function recovery at 4 weeks; i.v. EPO better than mannitol for renal I-R injury protection
2009	Rats	IR: transplanted with male bone marrow cells; reperfusion 2 or 4 weeks	EPO 5000 U 30 minutes before ischemia	↑GFR (4 weeks), →proteinuria/Hb (2 and 4 weeks), ↓tubulointerstitial changes
2008	Rats	IR: occlusion of infrarenal abdominal aorta, ischemia 30 min, reperfusion 60 min vs. sham	EPO 1000 U/kg s.c. 5 min before ischemia	↓MDA levels, ↓SOD activity, ↓catalase ↓histopathological changes
2007	Rats	IR: bilateral renal pedicle occlusion 1 day after last EPO injection, ischemia 45 min; reperfusion 24, 72 hr and 1 week	EPO 100 U/kg or 100 U/kg CEPO s.c. q. 2 days for 2 weeks vs. saline	CEPO: (no erythropoiesis) ↓apoptosis, ↓α-SMA expression, ↑tubular epithelial cell proliferation, ↓SCr. EPO: ↑Hb, ↓in apoptosis & α-SMA (not as marked as CEPO). CEPO more therapeutic than EPO.
2007	Rats	IR: bilateral renal pedicle occlusion 1 day after last EPO injection, ischemia 60 min; reperfusion 24, 72 hr	EPO 100 U/kg s.c. every 2 days for 2 weeks (6 injections) vs. saline	↑HIF-1alpha-positive cells, ↑VEGF mRNA expression, ↓tubular hypoxia, ↓apoptotic and α-SMA-positive interstitial cells
2008	Rats	IR: bilateral renal pedicle occlusion 1 day after last EPO dose, ischemia 45 min; reperfusion 24, 72 hr and 1 week	EPO or CEPO as above for 2 weeks	↑peritubular capillary endothelial cells. CEPO may protect kidneys from IR injury by promoting angiogenesis.
2007	Pigs	IR: unilateral nephrectomy; occlusion renal artery for 1 hr 1 week later, reperfusion 5 days	EPO 5000 U/kg IV at ischemia, then 1000 U/kg s.c. vs. no treatment	↓renal dysfunction, ↓cell death (histology at 5 days)
2006	Rats	IR: bilateral renal pedicle occlusion, ischemia 45 min; reperfusion 48 hr	EPO 500 U/kg i.p. 20 min before ischemia	↓SCr, ↓urea, ↓histological injury, ↓tubular apoptosis
2006	Rats	IR: bilateral renal pedicle occlusion, ischemia 45 min; reperfusion 1-7 days vs. sham/vehicle	EPO 5000 U/kg or DPO 25 μg/kg i.p. at time of ischemia or 6 hr after reperfusion	EPO & DPO at T0 and T6 -↓tubular apoptosis, ↓plasma creatinine/urea, ↑tubular regeneration (cell proliferation and mitosis)
2005	Rats	IR: R nephrectomy, clamp L pedicle 45 min and reperfusion 45 min and 24 hr	EPO 1000 U/kg and genistein (tyrosine kinase inhibitor) 10 mg/kg 2 hr before ischemia	↓SCr, ↓urea, ↓TNF-α and IL-2 expression (proinflammatory mediators of I-R injury), ↓LDH (indicates lipid peroxidation), ↓histological injury; genistein reversed benefits of EPO
2004	Rats	IR: uni/bilateral renal artery occlusion, 30 min ischemia, 24 or 48 hr reperfusion vs. sham/vehicle	EPO 5000 U/kg i.p. 30 min before ischemia	↓apoptosis, ↑regeneration, ↓casts, ↓plasma creatinine *in vitro*: ↓apoptosis, ↑mitosis/DNA synthesis
2004	Mice	IR: bilateral renal artery occlusion, 30 min ischemia, 24 hr reperfusion vs. sham/vehicle	EPO 1000 U/kg/day s.c. 3 days before I-R, or EPO 1000 U/kg s.c. on reperfusion	↓plasma creatinine, ↓plasma AST, ↓histological injury, ↓kidney MPO and MDA levels
2004	Rats	IR: bilateral renal pedicle occlusion, 45 min ischemia, 6 hr reperfusion vs. sham/saline	EPO 300 U/kg i.v. 30 min before ischemia, 5 or 3 min before reperfusion	↓tubular apoptosis, ↓tubular (NAG) and reperfusion (AST) injury, ↓histological injury, ↓SCr, better urine flow, ↑creatinine clearance
2004	Rats	IR: bilateral renal artery occlusion, 40 min ischemia, 48 (1) or 96 (2) hrs reperfusion vs. sham/vehicle	1. EPO 200 U/kg i.p. at start. 6 and 24 hr after reperfusion; or 200 U/kg i.v. and 4, 10, 24, 48 hr after reperfusion	↓plasma creatinine, ↓polyuria, ↓FENa, ↑AQP/NHE/TSC expression (prevented down-regulation of AQPs and Na+ transporters)
2003	Rats	IR: bilateral renal artery occlusion, 45 min ischemia, ≤72 hr reperfusion vs. sham/saline	EPO 3000 U/kg 24 hr pre I-R injury	↓SCr, ↓tubular necrosis, ↓tubular apoptosis, ↓tubular cell proliferation ↑bcl-2 protein, ↓caspase 3 activity, ↓JNK expression, dose-dependent
2001	Rats	IR: R kidney occlusion, 30 or 45 min ischemia, simultaneous L nephrectomy, ≤ 96 hr reperfusion vs. sham/vehicle	EPO 500 or 3000 U/kg i.v. at ischemia then s.c. 24, 48 hr after	↑HCt, ↓ mortality (severe ischemia group), →SCr/weight

IR = ischemia-reperfusion, ↑ = increased, ↓ = decrease(d), → = the same level, MDA = malondialdehyde, SOD = superoxide dismutase (an antioxidant), CEPO = carbamylated EPO, α-SMA = alpha-smooth muscle actin (associated with renal injury), SCr = serum creatinine, HIF-1 alpha = hypoxia inducible factor-alpha, VEGF = vascular endothelial growth factor, mRNA = messenger RNA, DPO = darbepoietin, R = right, L = left, TNF-α = tumor necrosis factor-alpha, IL-2 = interleukin-2, LDH = lactate dehydrogenase, PTC = proximal tubule cell, AST = aspartate aminotransferase (indicates reperfusion injury), MPO = myeloperoxidase, NAG = N-acetylglutamate, FENa = fractional excretion of Na+, AQP = aquaporin, NHE = Na+/H+ exchanger, TSC = thiazide-sensitive sodium chloride cotransporter, bcl-2 = oncogene activated by chromosome translocation in human B-cell lymphomas, JNK = c-Jun N-terminal kinase, HSP70 = heat shock protein 70

Each EPO molecule has two EPO receptor (EPOR) binding sites. There are two affinities of the EPOR for EPO in solution: one of high and one of low affinity (needs 1,000 times the concentration of EPO for activation) [8].

### Physiological stimuli for EPO production/release

Approximately 90% of systemic EPO in adults is produced by peritubular interstitial fibroblasts in the renal cortex and outer medulla of the kidney. A feedback mechanism involving oxygen delivery to the tissues appears to regulate EPO production [9]. Hypoxia-inducible factor (HIF) regulates transcription of the EPO gene in the kidney, which determines EPO synthesis. This process is dependent on local oxygen tension. HIF is quickly destroyed in well-oxygenated cells through ubiquitylation (tagging for degradation in the proteasome) by the von Hippel-Landau tumor suppressor protein (pVHL), but when oxygen delivery decreases, pVHL ceases its proteolysis of HIF, increasing the levels of HIF, which subsequently increases EPO production [10,11].

### Structure of EPO receptors

The EPO receptor (EPOR) is a 66 kD membrane glycoprotein typically consisting of 484 amino acids and 2 peptide chains; it belongs to a large cytokine and growth factor receptor family [3]. Binding studies have demonstrated that the EPOR has different affinities for EPO and that EPOR isoforms with higher affinity for EPO may be responsible for the erythropoietic effects of EPO, whereas isoforms with a lower affinity for EPO binding may have nonerythropoietic effects, such as tissue protection [12].

**Figure 1:**
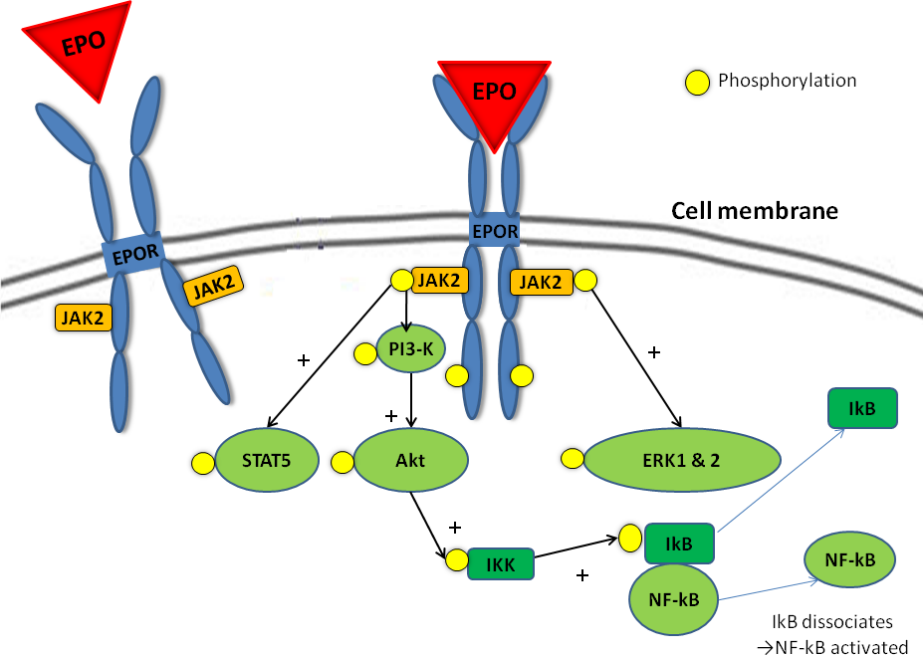
**The main pathways of the effects of EPO**. The intracellular domain of the EPOR contains phosphotyrosines, which are phosphorylated by activation of a member of the Janus-type protein tyrosine kinase family (JAK2) bound to the EPOR. These phosphotyrosines serve as docking sites for signal transducer and activator of transcription 5 (STAT5) and activate the mitogen-activated protein kinase (MAPK), phosphatidylinositol 3-kinase (PI3K), and protein kinase B (Akt) pathway. Akt stimulates IKK to phosphorylate, which phosphorylates the inhibitory IkB protein causing it to dissociate from NF-kB, which causes its activation (Modified from Anaesth Intensive Care 2011, in press).

The cytoplasmic domains of the EPOR contain a number of phosphotyrosines that are phosphorylated by the activation of a member of the Janus-type protein tyrosine kinase family (JAK2), which is bound to the common beta subunit of the EPOR [13]. In addition to activating the mitogen-activated protein kinase (MAPK), phosphatidylinositol 3-kinase (PI3K), and protein kinase B (Akt) pathway (Figure 1), phosphotyrosines also serve as docking sites for signal transducer and activators of transcription (STATs), such as STAT5. Dephosphorylation of JAK can be induced by phosphatase with the consequent internalization and degradation of the EPO/EPOR complex, which marks the end of EPO activity. This prevents overactivation, which may lead to excessive erythrocytosis [14].

### Post-receptor (intracellular) effects of EPO

There are a number of common pathways through which EPO exerts its erythropoietic effects that also appear to confer tissue protection. EPO "classically" binds to two EPORs, which become joined as a homodimer and change. This activates JAK2, which is bound to the common beta subunit of the EPOR [13] and leads to phosphorylation of tyrosine residues of the EPOR, which activates a number of signaling pathways (Figure 1).

EPO classically signals through the "signal transducer and activator of transcription 5" (STAT-5) pathway. The STAT proteins are direct substrates of Janus kinases (JAKs), which results in tyrosine phosphorylation of the STATs as well as phosphorylation of the phosphatidylinositol 3-kinase (PI3K) and subsequent phosphorylation of Akt (Figure 1).

**Figure 2:**
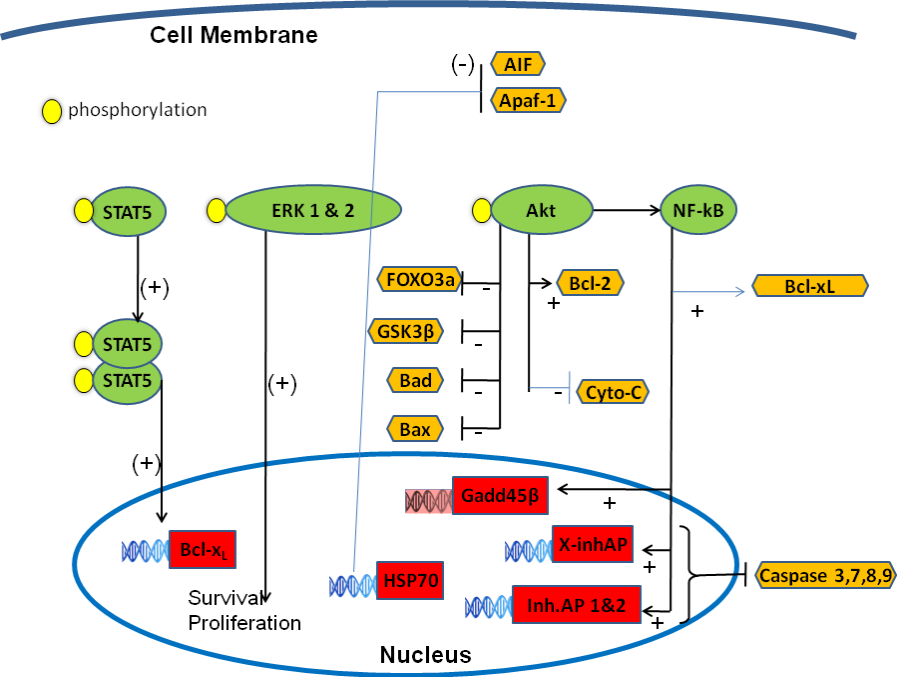
**Apoptotic pathways influenced by EPO**. Activated STAT5 promotes transcription of promitogenic and antiapoptotic genes associated with apoptotic regulation and cytoprotection. Akt promotes cell survival and antiapoptotic effects by 1) inhibiting forkhead transcription factor (FOXO3a), a trigger for apoptosis; 2) inactivating glycogen synthase kinase 3β (GSK3β), thus preventing cell injury; 3) reducing the activity of proapoptotic proteins Bad and Bax; 4) increasing the activity of antiapoptotic protein Bcl-2; 5) preventing cytochrome C release. NF-kB prevents apoptosis by 1) inducing expression of the inhibitors of apoptotic protein (inh. AP 1&2) and the x-chromosome-linked IAP (X-InhAP), which inhibits caspase 3, 7, and 9; 2) promoting induction of growth arrest and DNA damage protein (Gadd45β), which is associated with cell cycle and DNA repair and suppression of apoptosis; and 3) by activating Bcl-x_L_. EPO induces heat shock protein 70 (HSP70), which inhibits AIF moving into the nucleus, thus avoiding DNA fragmentation and apoptosis. HSP70 also prevents Apaf-1/cytochrome C (Cyto-C) binding, a complex involved in proapoptotic caspase activation. NB: the + sign indicates activation and the - sign indicates inhibition. (Modified from *Anaesth Intensive Care *2011, in press).

The principal component of pathways that promote anti-apoptotic effects is **Akt **(Figure 2), which inactivates caspases, the major mediators of apoptosis, mitochondrial dysfunction, and subsequent release of cytochrome C [15]. EPO's ability to maintain cellular integrity and prevent inflammatory apoptosis is closely linked to maintenance of mitochondrial membrane potential, modulation of Apaf-1, inhibition of cytochrome C release, and inhibition of caspases. Recent data also indicate that serum and glucocorticoid-regulated kinase-1 (SGK1) may contribute to the mediation of EPO's renoprotective effects [16].

The phosphorylation of mitogen-activated protein kinases (MAPKs) appears to contribute to the cell protection EPO confers (Figure 2). Protein kinase C (PKC) also is involved in inhibition of apoptosis and cell survival. It regulates the EPO-induced erythroid proliferation and differentiation [17] and interferes with phosphorylation of the EPOR, making it a likely upstream modulator of the EPOR.

EPO may be involved in modulation of cellular calcium homeostasis by increasing calcium influx [18]. Nuclear factor-kappaB (NF-kB), a mediator of inflammatory and cytokine response, is implicated in EPO signaling. The cytoprotection of EPO partly depends on Akt and subsequent NF-kB activation (Figures 1 and 2). NF-kB plays a role in the release of EPO during HIF-1 induction; Akt can increase NF-kB and HIF-1 activation with resultant increase in EPO expression [19].

Finally, induction of heat shock protein 70 (HSP70) by EPO is related to renal protection in ischemic kidneys [20]. HSP70 prevents apoptosis by inhibiting movement of apoptosis inducing factor (AIF) to the nucleus [21] and by preventing Apaf-1/cytochrome C binding in the cytosol [22] (Figure 2).

### Red blood cell effects of EPO

The principal physiological function of EPO is red blood cell production, which results from a tightly controlled proliferation and differentiation pathway [23]. Early hematopoietic progenitor cells differentiate into burst-forming unit-erythroid cells (BFU-Es). Continuous stimulation with EPO triggers the differentiation of CFU-Es into erythroblasts, which lose their nuclei to form reticulocytes. After a few days, reticulocytes lose reticulin and become erythrocytes (red blood cells). Reticulocytes and erythrocytes stop expressing EPOR and cease being responsive to EPO [24].

EPO-binding to EPORs on erythroid progenitor cells leads to activation of the JAK2-STAT5 signaling pathway and phosphorylation of PI3K and Akt1 [25] (Figure 1). Akt-mediated phosphorylation of Bad in the Bad-Bcl-xL complex releases the antiapoptotic protein Bcl-xL, which suppresses erythroid progenitor cell apoptosis [26]. Akt also is involved in several pathways that promote cell survival and antiapoptotic effects through inhibition of FOXO3a, inactivation of GSK3β, induction of XIAP, inactivation of caspases, and prevention of cytochrome C release (Figure 2). These effects not only enhance the erythropoietic properties of EPO but appear to be important in the protection of other cell types and may contribute to the reported neuronal and renal protective effects [25].

## The pleiotropic effects of EPO

The tissue protective or "pleiotropic" effects (from the Greek, πλείων - *pleion*, meaning "more," and τρέπειν - *trepein *meaning "turn or convert") of EPO beyond erythropoiesis have been shown in the kidney in many animal and some clinical studies.

Its tissue protective effects may be elicited through the EPOR homodimer via JAK2-STAT5 activation and inhibition of apoptosis or may be mediated by a second EPOR isoform heterodimer composed of an EPOR monomer and the cytokine receptor, common beta subunit (CD-131). For example, carbamylated EPO (CEPO) does not bind to the classical EPOR isoform and is devoid of hematopoietic activity; however, it can provide tissue protection in the kidney, supporting the existence of a heteroreceptor EPO isoform, which mediates tissue protection [27]. It is clear that the relationship of EPO with its receptor is extremely complex. Therefore, further investigation is required to fully understand the EPOR heterodimer isoform, and the mechanisms and pathways involved in its tissue protective activity.

### Renal protection

#### Animal and in vitro studies

Many animal studies have shown that EPO administration protects kidney tissue from damage and improves renal function in ischemia-reperfusion (IR) and contrast-induced injury models of AKI (Tables 1 and 2) [25,28-52] in which EPO reduced kidney dysfunction by decreasing apoptosis. In addition, EPO has been shown to reduce the expression of proinflammatory mediators, TNF-alpha and IL-2, in IR renal injury and reverse the effect of endotoxin on the antioxidant, renal superoxide dismutase (SOD). These anti-inflammatory properties of EPO also suggest involvement of the NF-kB pathway in its kidney protection.

**Table 2:** Animal studies of EPO in nonischemic models of AKI

Year	Type	AKI model	EPO dose	Outcome
2010	Rats	Brain death + Perfused kidney model	10 μg/kg EPO or CEPO IV, 4 hr brain death, then kidney reperfusion in perfused kidney model	EPO and CEPO: ↓expression of proinflammatory genes, ↓infiltration of polymorphonuclear cells in kidney, preserved vascular integrity. CEPO more effective than EPO. Kidney function fully restored with EPO and CEPO.
2010	Mice	Aristolochic acid nephropathy	DPO 0.1 mcg/kg wkly from day of Aristolochic acid administration or on day 28	↑survival of tubular cells lead to ↓acute tubular injury, interstitial inflammation and interstitial fibrosis.
2008	Rats	CIN: Ioversol 2.9 g/kg iodine + inhibition of prostaglandin and NO synthesis	EPO 10,000 U/kg or asialoEPO 80 ng/g i.v. 1 hr before Ioversol	↓renal dysfunction and histological injury, ↓apoptosis, ↓caspase3-activated apoptosis in renal porcine epithelial cells in vitro with ↑JAK2/STAT5 phosphorylation and HSP70 expression; ↑JAK2/STAT5 phosphorylation and HSP70 expression in rat kidneys *in vivo*.
2008	Rats	*in vitro */CIN: cisplatin 5.5 mg/kg i.v.	EPO 5,000 U/kg i.v. OR equivalent peptide mass of inactive EPO OR DPO 25 μg/kg before cisplatin OR saline	EPO ↑HCt, ↓SCr; DPO also ↑HCt, ↓SCr. Clearance studies: GFR and renal blood flow confirmed DPO renal protection. ↓tubular apoptosis and necrosis with DPO. DPO 48 hr after cisplatin was renoprotective.
2008	Mice	CIN: i.p. cisplatin injection (10 mg/kg/day) for 2 days vs. placebo. Follow-up 6 days.	EPO 1,000 U/kg i.p. daily ≤ 3 days before cisplatin vs. vehicle	↓urea, ↓casts, ↑marrow stem cell (MSC) numbers
2007	Mice	Endotoxemia: 2.5 mg/kg endotoxin i.p. (lipopolysaccharide); follow-up 16 hrs later	EPO 4000 U/kg 30 min before endotoxin vs. vehicle	↑GFR (inulin clearance), →MAP, →Renal bld flow, ↑CRP, →serum NO, EPO reversed the endotoxin effect on renal SOD activity (SOD ↓ in control group).
2006	Rats	CIN: (iothalamate), following indomethacin and Nω nitro-L-arginine methyl ester	EPO 3000 U/kg and 600 U/kg i.v. 24 and 2 hr pre-CIN induction vs. saline	Creatinine clearance preserved
2005	Rats	Chronic kidney disease	DPO s.c. 0.4 μg/kg/wk into 5/6 remnant kidney rats after renal mass reduction	↑microvascular density, ↑endothelial proliferation, preserved renal function (↓SCr), ↓scarring, ↑VEGF expression
2004	Rats	Hemorrhagic shock and endotoxic shock	EPO 300 U/kg i.v. before resuscitation	↓renal dysfunction in hemorrhagic but not endotoxic shock
2001	Rats	CIN: cisplatin toxicity - i.p. cisplatin injection 6 mg/kg vs. placebo	EPO 100 U/kg i.p. before cisplatin, then daily for 9 days vs. placebo	↑renal blood flow/GFR at 9 days, ↑ tubular regeneration, ↑tubular cell proliferation, ↑functional recovery
1994	Rats	CIN: cisplatin toxicity - i.p. cisplatin injection 7 mg/kg vs. placebo	EPO 100 U/kg i.p. after cisplatin, then daily for 9 days vs. placebo	↑functional recovery, ↑tubular regeneration, ↑DNA synthesis

CEPO = carbamylated EPO, DPO = darbepoietin, ↑ = increased, ↓ = decrease(d), → = same level, H_2_O_2 _= hydrogen peroxide, JAK2/STAT5/Akt = signaling pathways of EPO, GSK-3β = glycogen synthase kinase 3 beta, mTOR = mammalian target of rapamycin, ERK = extracellular signal-regulated kinase, CIN = contrast-induced nephropathy, NO = nitric oxide, HSP70 = heat shock protein 70, RPTE = renal proximal tubular epithelial, SCr = serum creatinine, MAP = mean arterial pressure, SOD = superoxide dismutase (an antioxidant), VEGF = vascular endothelial growth factor

CEPO: The administration of carbamylated EPO (CEPO), which does not bind to the classical EPOR, also provides renal tissue-protective effects. In an IR rat model, CEPO markedly reduced apoptosis and increased tubular epithelial cell proliferation. Moreover, CEPO was more protective against IR injury to tubular epithelial cells than EPO in this study. In an *in vitro *model performed by the same team, CEPO promoted more capillary formation than EPO and also appeared to protect the kidneys from IR injury by promotion of angiogenesis [52]. This protective effect requires mitogenesis and endothelial progenitor cell differentiation, proliferation, and migration.

EPO activates endothelial nitric oxide synthase, and this effect on the endothelium may be critical for the renal tissue protective effects of EPO. EPO is an extremely potent stimulator of endothelial progenitor cells, whose function is partly dependent on nitric oxide bioavailability. Endothelial progenitor cells appear to be involved in endothelial recovery after injury. EPO limits AKI in part by stimulating vascular repair and by mobilizing endothelial progenitor cells and increasing tubular cell proliferation [27]. These findings suggest that EPO may exert a protective effect via an interaction with the microvasculature.

Angiogenesis and EPO's renoprotective effects may be influenced by vascular endothelial growth factor (VEGF). Nakano and colleagues found that the vascular EPO/EPOR system promoted postischemic angiogenesis by upregulating the VEGF/VEGF receptor system, both directly by promoting neovascularization and indirectly by mobilising endothelial progenitor cells and bone marrow-derived proangiogenic cells [53]. It appears that angiogenesis is impaired and blood vessels are less responsive to VEGF in the absence of EPOR.

### EPO in AKI

AKI is common in critically ill patients [54,55] and is independently associated with increased mortality, and with prolonged length of stay. It escalates both the human and financial costs of care. Therefore, it seems desirable to investigate treatments with potential to ameliorate or prevent AKI.

Some injury pathways for AKI in the critically ill include exposure to endogenous and exogenous toxins, metabolic factors, ischemia and reperfusion insults, neurohormonal activation, inflammation, and oxidative stress. Of these, ischemia-reperfusion may be the most common. EPO can prevent or reduce injury and assist renal repair and recovery through limitation of apoptosis, promotion of neovascularization, anti-inflammatory action, and tissue regeneration.

Investigation of potential treatments for AKI has had limited success to date; however, from the results of animal and some limited preliminary human studies, therapeutic use of EPO seems promising for those "at risk" for AKI.

### Clinical trials of EPO in AKI

One randomized, placebo-controlled, clinical trial of preoperative EPO in 71 patients who underwent elective coronary artery bypass graft (CABG) surgery reported renoprotective effects [56] (Table 3). EPO was given at a dose of 300 U/kg IV immediately preoperatively and was associated with a reduction in the incidence of AKI from 29% to 8% (*p *= 0.035) and improved postoperative renal function as indicated by a smaller increase in SCr (% increase at 24 hours of 1% vs. 15%, *p *= 0.04) and a smaller decline in estimated GFR (% change at 24 hours of +3% vs. -5%, *p *= 0.04) postoperatively (Table 3).

**Table 3:** Comparison EPO in CABG surgery vs. EARLYARF trials

	EPO in CABG	EARLYARF
Sample size	71 (EPO: 36, placebo: 35)	162 (EPO: 84, placebo: 78)
Patient population	Elective CABG	Aim: ICU patients at high risk of AKI; Obtained: critically ill patients
Study design	Prospective randomised double-blind, placebo-controlled trial	Prospective randomised double-blind placebo-controlled, trial
EPO type and dose	1 dose preop: 300 U/kg EPO or normal saline IV	2 doses: EPO-beta 500 U/kg to max 50,000 U or normal saline IV
Inclusion criteria	> 18, elective CABG	↑ in GGT and ALP urine concentration product > 46.3
Exclusion criteria	Emergent CABG, pre-existing AKI, on RRT, uncontrolled HT, nephrotoxic drugs within 3 days of op, previous use of EPO	< 16 yr, no IDC, hematuria, rhabdomyolysis, myoglobinuria, polycythemia, cytotoxic chemo, RRT or needs in 48 hr, stay ≤24 hr, survival ≤72 hr, prior RIFLE "failure"
Measurements	Baseline SCr preop and 24, 72, and 120 hr postop	Baseline creatinine: various versions of preop/pre-ICU creatinine, including lowest on ICU admit/last ICU creatinine/minimum at 12 mo. Blood for creatinine and Cyst C and start for 4/24 creatinine clearance
Age (mean)	66.7	61.6
Study groups: EPO vs. placebo	Baseline and intraoperative: no significant differences, most OPCABG (77%) (+3valves in EPO group)	EPO group older (*p *= 0.011) and ↑ likelihood for sepsis (*p *< 0.05). More placebo patients had AKI (not significant)
Primary outcome	Incidence of AKI after CABG	*A priori: *Average % plasmaCr↑ from baseline over 4-7 days.
Secondary outcomes	Changes in SCr and eGFR (first 5 days postop), ICU and hospital LOS, in-hospital mortality	AKIN & RIFLE AKI definitions, plasma cystatin C, need for dialysis, death within 7/30/90 days;
AKI: Definition	≥50%↑ in SCr from preop baseline	AKIN (creatinine and UO) and RIFLE (creatinine) definitions
AKI: Proportion	EPO 8%, placebo 29%; *p *= 0.035	AKIN creatinine: EPO 45.2%, placebo 47.4%; RIFLE creatinine: EPO 23.8%, placebo 19.2%; AKIN UO: EPO 70.2%, placebo 51.3% (*p *= 0.016)
Results	%SCr↑ at 24 hr: EPO 1 ± 3, placebo 15 ± 7 (*p *= 0.04). %SCr↑ at 120 hr: EPO 7 ± 4, placebo 27 ± 8 (*p *= 0.01). %eGFR↓ at 24 hr: EPO 3 ± 3, placebo -5 ± 4 (*p *= 0.04). %eGFR↓ at 120 hr: EPO -4 ± 3, placebo -13 ± 5 (*p *= 0.01)	No significant difference in 1° outcome or 2° outcomes except AKI (AKIN UO). Of randomised pts without AKI initially (n = 104) EPO patients had higher %plasmaCr↑: EPO 8.5 ± 27(n = 61), Placebo -4.6 ± 18 (n = 47; *p *= 0.004).
Onset of injury	Initiation of CABG operation	Heterogenous. Time of injury estimated for subdivision analysis. Samples from 6-12 hr after putative insult more predictive for AKI (AUC = 0.69), dialysis, and death.
AKI mechanism	CVS compromise, CPB exposure	Heterogenous
Safety	No symptomatic thrombosis or other adverse events in EPO patients	No evidence for ↑intravascular thrombosis. EPO not associated with ↑ in adverse events.

CABG = coronary artery bypass graft, GGT = γ-glutamyl transpeptidase, ALP = alkaline phosphatase, chemo = chemotherapy, pre/post/intraop = pre/post/intraoperative(ly), RRT = renal replacement therapy, HT = hypertension, IDC = indwelling catheter, RIFLE and AKIN = AKI classification systems, (S)Cr = (serum) creatinine, Cyst C = cystatin C, OPCABG = off pump CABG, ICH = intracranial hemorrhage, LOS = length of stay, UO = urine output criteria, CVS = cardiovascular system, CPB = cardiopulmonary bypass, I-R = ischemia reperfusion

A more recent and larger (n = 162) study (EARLYARF trial) assessed EPO's effect in ICU patients deemed at risk for AKI (defined by a specific cutoff value of two proximal tubular enzymes in urine: γ-glutamyl transpeptidase [GGT] and alkaline phosphatase [ALP]) (Table 3) [57]. EPO was given at 500 U/kg IV when a high GGT × ALP product was detected and repeated 24 hours later. The primary outcome of this study was the average percent creatinine increase from baseline to its peak over 7 days. Unlike the study in CABG patients, this trial found no renoprotective effect of EPO. The reasons for these contradictory findings may be related to the differences in design and methods used.

### EARLYARF trial

The EARLYARF trial is the largest trial of EPO as a nephroprotective agent. However, it suffered from several important methodological limitations. First, it used poorly validated biomarkers as predictors of high risk for AKI. This approach, along with an untested cutoff value to trigger participant inclusion, resulted in the inclusion of a general critically ill cohort rather than only those at true high risk for AKI. The transient increase in urinary GGT and ALP seen in this trial, along with the uncertainty about the timing of the insult in a heterogeneous cohort, limited the ability to draw conclusions about the efficacy of early EPO administration. Importantly, the biomarkers selected to trigger protection against AKI by means of EPO therapy were very poor in predicting AKI with a receiver operating characteristic area under the curve of only 0.54. The decision to randomize was based solely on these biomarkers and did not take into account additional clinical factors that might assist in determining the risk of AKI.

A second problem in this study relates to the inclusion of patients with likely volume-responsive prerenal AKI, which would likely reduce the study's power, to determine the true efficacy of EPO. Fractional sodium excretion was low in most randomized patients, suggesting intact renal sodium reabsorption. The exclusion or identification and separate treatment of patients with prerenal AKI may reduce their confounding effect on results.

Third, serum creatinine measurements used for the baseline in the primary outcome were based on inconsistent criteria and timing. Some were taken before or around the time of ICU admission and others after adding additional confounders to the analysis. For the vast majority of patients, no creatinine measurement was available. Thus, the diagnosis of AKI was based on a retrospective (post-hoc) analysis using different criteria (lowest creatinine on ICU admission, last ICU value, lowest value at follow-up). These varying criteria can influence the accuracy of any assessment.

Fourth, at randomization, approximately one third of patients had already met the criteria for AKI, suggesting that the intervention was relatively late in the process of injury for many. In addition, the trial size was small with clear limitations in statistical power.

Fifth, in patients with early AKI (> 30%), measurement of the reduction in AKI stage and improved recovery should replace the use of development of AKI as an endpoint. RIFLE and AKIN classification systems can be used in this way.

Sixth, there were baseline differences between the study groups. For example, the EPO group were older and more likely to have sepsis (*p *< 0.05).

As discussed by the investigators, these limitations make it impossible to use this study to exclude a potential therapeutic renoprotective effect of EPO.

### Potential risks of EPO

#### Pure red cell aplasia

Despite the numerous benefits of EPO, there are some risks. Pure red cell aplasia is a rare adverse event, which is characterized by anemia, low reticulocyte count, absence of erythroblasts, resistance to EPO, and neutralizing antibodies against EPO [58]. This is an extremely rare complication.

#### Cancer patients

EPO administration in patients with cancer has been associated with increased mortality and enhanced tumor growth [59]. The underlying mechanisms remain uncertain, but patients with certain malignancies may be in a hypercoagulable state, making EPO administration unadvisable.

#### Thrombosis

Recent studies and clinical trials have found an increased rate of thrombosis with EPO, which has mainly been observed in patient groups with higher than conventional levels of hemoglobin (> 120 g/L). Exclusion of patients with hemoglobin >120 g/L from clinical trials of EPO minimizes the risk for thrombosis. Nonetheless, systematic assessment for thrombosis should be performed in any EPO trials of critically ill patients because they have an increased risk for thrombosis.

#### Hypertension

Hypertension occurs in approximately 30% of patients who receive long-term EPO treatment and appears to involve increased endothelin release, upregulation of tissue renin and angiotensin production, changes in the balance of vasoactive substances (prostaglandin/prostacyclin/thromboxane), and an elevation of calcium by EPO (at least in chronic kidney disease) that impairs the vasodilating action of nitric oxide. It is advisable that patients with uncontrolled hypertension do not participate in trials of EPO in AKI.

Carbamylated EPO, a cytoprotective, nonerythropoietic derivative of EPO may not exhibit the same risks as EPO and holds great interest as a future tissue-protective therapy. However, it requires further experimental testing before it can be safely evaluated in clinical trials.

## EPO-AKI

EPO-AKI and EPO-Biomarkers are the two components of the renal substudy of the EPO-TBI trial (NCT00987454). EPO-TBI is a trial of EPO as cerebral protection after traumatic brain injury, and the renal substudy assesses the effect of EPO on the development of acute kidney injury (AKI) and the response to treatment using multiple renal biomarkers with different time profiles in patients with traumatic brain injury. Having recently sustained a timed physical injury, this homogenous cohort lends itself to such a study. AKI will be classified using methods based on the RIFLE criteria. Baseline renal function will be taken from a consistent source for all patients. With a cohort of 606 patients, this will be the largest study of EPO to protect against AKI ever performed, increasing the probability of detecting a treatment effect. Furthermore, it will be the first EPO trial to incorporate active risk assessment for thrombotic episodes. Weekly intervals separate the doses of EPO (up to 3) in this trial to allow time for clearance and to avoid excessively high levels of EPO. In addition, patients with a known malignancy and/or uncontrolled hypertension will be excluded, thus minimizing risk to patients. In a paradoxical way, the EPO-TBI trial provides a unique opportunity to clarify the potential benefit of EPO as a kidney protective agent. This trial also may provide valuable insight into the mechanisms of EPO in AKI and pave the way for further dedicated large scale trials of EPO in AKI.

## Conclusions

Many experimental studies and a strong biological rationale support the notion that EPO may be an effective nephroprotective drug. Only two studies have assessed its efficacy in this regard: one study of patients who received elective coronary artery bypass grafting, and the other in a heterogeneous group of critically ill patients. The first showed benefit; the second did not. Thus, there is uncertainty as to whether the experimental benefits can be translated into clinical benefits. A study of EPO in TBI, however, is now underway and will randomize 606 patients to placebo or EPO. This study will offer a unique opportunity to study, in a larger and more homogeneous cohort, whether EPO does confer renal protection in patients at risk of AKI. Until such data are available or other studies emerge to provide more clinical data, EPO remains an attractive but unproven nephroprotective agent.

## Competing interests

RB is an investigator of a National Health and Medical Research Council/Victorian Neurotrauma Initiative funded clinical trial of Erythropoietin in Traumatic Brain Injury (NCT00987454).

## Authors' contributions

EM wrote the first draft of the manuscript. RB reviewed and modified the initial draft.
